# N-terminal pro-B-type natriuretic peptide is associated with clinical outcomes after transcatheter aortic valve replacement

**DOI:** 10.1186/s13019-023-02391-2

**Published:** 2023-10-10

**Authors:** Jun Yu, Wei Wang

**Affiliations:** Department of Structural Heart Disease Centre, National Center for Cardiovascular Diseases, Fu Wai Hospital, Chinese Academy of Medical Sciences and Peking Union Medical College, A 167, Beilishi Road, Xicheng District, Beijing, 100037 China

**Keywords:** Aortic valve disease, NT-proBNP, Biomarker, Transcatheter aortic valve replacement, Risk stratification

## Abstract

**Background:**

Limited data on the prognostic value of periprocedural changes of plasma N-terminal pro-B-type natriuretic peptide (NT-proBNP) after transcatheter aortic valve replacement (TAVR).

**Methods:**

Data of plasma NT-proBNP were retrospectively collected in 357 patients before TAVR procedure and at discharge from January 1, 2018 to December 31, 2021 in our single center. Patients were grouped as responders and non-responders according to the NT-proBNP ratio (postprocedural NT-proBNP at discharge/ preprocedural NT-proBNP). Responders were defined as NT-proBNP ratio < 1 and non-responders were defined as NT-proBNP ratio ≥ 1. Outcomes were defined according to the Valve Academy Research Consortium (VARC)-3 criteria.

**Results:**

A total of 234 patients (65.5%) and 123 patients (34.5%) were grouped as the responders and the non-responders, respectively. Responders and non-responders were significantly different in both median preprocedural (2103.5 vs. 421.0 pg/ml, p < 0.001) and postprocedural (707.6 vs. 1009.0, p < 0.001) NT-proBNP levels. Patients in the non-responder group were more inclined to have comorbidities of hypertension (73.2% vs. 51.7%, p < 0.001), hyperlipidaemia (46.3% vs. 34.6%, p = 0.031), peripheral vascular disease (20.3% vs. 8.5%, p = 0.001) and pure aortic insufficiency (15.4% vs. 4.3%, p < 0.001). In the contrast, patients in the responder group had higher prevalence of maximum transvalvular velocity (4.6 vs. 4.2 m/s, p < 0.001), reduced left ventricular ejection fraction (58.0% vs. 63.0%, p < 0.001), heart failure (9.4% vs. 2.4%, p = 0.014), mitral regurgitation ≥ moderate (13.7% vs. 4.9%, p = 0.010), tricuspid regurgitation ≥ moderate (12.0% vs. 2.4%, p = 0.002), and pulmonary hypertension (32.9% vs. 13.0%, p < 0.001). Patients in the non-responder group were moderately longer than the responder group in total hospitalization length (14 vs. 12 days, p < 0.001). The non-responder group were significantly associated with cumulative all-cause mortality (p = 0.009) and cardiac mortality (p < 0.001) during the follow-up period.

**Conclusions:**

Periprocedural changes of NT-proBNP is clinically useful for the risk stratification of survival in patients after TAVR.

**Supplementary Information:**

The online version contains supplementary material available at 10.1186/s13019-023-02391-2.

## Introduction

Transcatheter aortic valve replacement (TAVR) has emerged as a safe and effective treatment for severe aortic stenosis [1]. A growing number of patients are being referred for TAVR due to the expanding indications for the procedure [2]. It is necessary to develop improved tools for risk stratification of this patient group. Researchers have investigated a variety of biomarkers such as high-sensitive troponin T, GDF15 (growth differentiation factor 15), osteopontin, and soluble ST2 (suppression of tumorigenicity 2) to determine the prognosis in TAVR patients. As a result, more biomarkers are required to predict treatment outcomes and stratify risks.

The N-terminal pro-brain natriuretic peptide (NT-proBNP) is an inactive derivative of cerebral natriuretic peptide (BNP) and has a half-life of 120 min [3, 4]. NT-proBNP is released in response to myocyte stretching following left ventricular hypertrophy [5]. Hence, monitoring NT-proBNP levels may assist in identifying individuals at high risk of adverse cardiovascular events such as left ventricular systolic dysfunction, left ventricular hypertrophy, and heart failure [6–8]. Both BNP and NT-proBNP have been identified of predictive value of prognosis in patients undergoing aortic valve replacement. In TAVR patients with aortic stenosis, elevated BNP and NT-proBNP pre- and post-TAVR were associated with clinical outcomes in many studies. However, these studies all excluded patients with pure native aortic regurgitation. The periprocedural changes of plasma NT-proBNP levels and the precise cut points for NT-proBNP at discharge after TAVR are still being clarified.

We therefore investigated the association between NT-proBNP ratio (postprocedural NT-proBNP at discharge/ preprocedural NT-proBNP) and clinical outcomes in TAVR patients, as well as the precise cut points of NT-proBNP at discharge after TAVR in predicting the cumulative late all-cause mortality. These findings may raise concerns about optimizing the timing of TAVR or medical therapy after TAVR to mitigate the pathobiological changes caused by NT-proBNP, thereby improving clinical outcomes.

## Materials and methods

### Study population

A total of 358 Patients underwent TAVR from January 1, 2018 to December 31, 2021 at our institution and 357 patients were included in the present study (1 patient died the day after TAVR immediately for multiple organ failure). NT-proBNP levels were measured in all patients at baseline before TAVR and at discharge after TAVR. This study was approved by the Medical Ethics Review Committee of Fuwai Hospital and informed consent was waived for the nature of retrospective analysis.

### Transcatheter aortic valve replacement procedure

TAVR was performed in a hybrid operating room by the heart team, and patients were under general anesthesia or local anesthesia and monitored by anesthesiologists. All patients underwent TAVR after discussions with the multidisciplinary heart team, and the access site and the type of prosthesis were determined thereafter. Transfemoral access was preferred in all patients with criteria unless prosthesis size, calcification and atheroma of the aorto-iliofemoral artery were considered. All TAVR procedures were performed according to established standards via transfemoral, trans-carotid, trans-subclavian, and contemporary devices of SE-valves (Venus-A [Venus Medtech, Hangzhou, China], VitaFlow [Microport, Shanghai, China], TaurusOne [Peijia Medical, Suzhou, China]) [9–11] or balloon expandable (BE) valves (Sapien 3 [Edwards Lifesciences, Irvine, California], Sapien XT [Edwards Lifesciences, Irvine, California]) [12, 13] were implanted.

### Data collection

Plasma NT-proBNP have been routinely measured within 48 h before TAVR procedure and 4 to 8 days after TAVR at the date of hospital discharge. Blood samples were drawn from the antecubital or dorsal hand vein and collected in EDTA-containing tubes and immediately processed by the Institute of Clinical Chemistry at Fuwai hospital. NT-proBNP were analyzed from Roche Diagnostics by electro-chemiluminescence immunoassay. The effective measuring range of the assay was 5-35000 pg/ml (a normal value of < 150 pg/ml for the general population). Patients were considered as non-responders if their NT-proBNP value at discharge post-TAVR was equal or greater compared with the baseline value. Baseline characteristics, procedural, and hospitalization data were retrospectively recorded manually from institutional electronic medical record system and entered into a dedicated database. All data were anonymized, systematically collected and assessed for quality.

### Follow-up and end points

All endpoints in this study were defined in line with the Valve Academic Research Consortium (VARC)-3 criteria [14]. The primary end point of the study was late cumulative all-cause mortality after TAVR. Patients were followed up till May 31, 2022 by outpatient visits and telephone interview. The median follow-up time was 640.7 days (interquartile range [IQR]:413.7 to 1073.0 days).

### Statistical analysis

All continuous variables were tested for normality using the Shapiro-Wilk test. Continuous variables were presented as mean ± standard deviation (SD) and compared using the Student’s t-test, or medians (25th -75th quartile) compared using Mann-Whitney U test. Categorical variables were presented as numbers and percentages and compared using the Chi-square test or Fisher’s exact test. Cumulative clinical outcomes were calculated using the Kaplan-Meier survival analysis, and the log-rank test was used for comparison between the groups. To identify independent predictors of late all-cause mortality, all variables with a p value < 0.05 on univariate analysis were included in a stepwise multivariable Cox regression model. The proportional hazard assumption was confirmed by examination of log (-log [survival]) curves and by testing of partial (Schoenfeld) residuals, and no relevant violations were found. The estimated hazard ratio (HR) with 95% confidence interval (CI) was provided by the Cox regression analysis. To find the cut-off value of NT-proBNP at discharge (1350.5 pg/ml), receiver-operating characteristic (ROC) curve evaluation was performed to assess the area under the curve (AUC) at the 95% confidence interval (CI) in predicting cumulative all-cause mortality. And NT-proBNP ≥ 1350.5 pg/ml was included in the stepwise multivariable Cox regression model. The trend of cumulative all-cause mortality across quartiles of NT-proBNP levels at discharge was analyzed using the Cochrane-Armitage test. To identify the determinants of NT-proBNP non-responders, a multivariable logistic regression analysis was performed using the following covariates selected from factors that were significantly different between NT-proBNP responders and non-responders: hypertension, hyperlipidaemia, NT-proBNP ≥ 1429.60 at baseline (median of NT-proBNP at baseline), peripheral vascular disease, heart failure, pure aortic insufficiency, left ventricular ejection fraction, maximum velocity, mitral regurgitation ≥ moderate, tricuspid regurgitation ≥ moderate, pulmonary hypertension, total length of hospitalization. All tests were 2-sided, and p values < 0.05 were considered to be statistically significant. All statistical analyses were performed using SPSS software version 26.0 (IBM, NY, USA) and R software version 4.1.0 (available at http://www.r-project.org).

## Results

### Baseline patient characteristics

A total of 357 patients were included for analysis. According to NT-proBNP ratio, 234 patients were divided into responder group for NT-proBNP ratio < 1 and 123 patients were divided into non-responder group for NT-proBNP ratio ≥ 1. The main baseline characteristics of the study population are summarized in Table 1. The two groups did not differ significantly in terms of age (mean: 72.4 years), male sex (57.1%), body mass index (mean: 24.7 kg/m^2^), New York Heart Association functional class III or IV (70.3%), diabetes mellitus (23.5%), chronic obstructive pulmonary disease (3.6%), chronic kidney disease (3.4%), and coronary artery disease (38.7%). Responders and non-responders were significantly different in both median preprocedural (2103.5 vs. 421.0 pg/ml, p < 0.001) and postprocedural (707.6 vs. 1009.0, p < 0.001) NT-proBNP levels. However, patients in the non-responder group were more inclined to have comorbidities of hypertension (73.2% vs. 51.7%, p < 0.001), hyperlipidaemia (46.3% vs. 34.6%, p = 0.031), peripheral vascular disease (20.3% vs. 8.5%, p = 0.001) and pure aortic insufficiency (15.4% vs. 4.3%, p < 0.001). In the contrast, patients in the responder group showed significantly higher prevalence of maximum velocity (4.6 vs. 4.2 m/s, p < 0.001), reduced left ventricular ejection fraction (58.0% vs. 63.0%, p < 0.001), chronic heart failure (9.4% vs. 2.4%, p = 0.014), mitral regurgitation ≥ moderate (13.7% vs. 4.9%, p = 0.010), tricuspid regurgitation ≥ moderate (12.0% vs. 2.4%, p = 0.002), and pulmonary hypertension (32.9% vs. 13.0%, p < 0.001). Patients in the non-responder group were moderately longer than the responder group in total hospitalization length (14 vs. 12 days, p < 0.001).

**Table 1:** Baseline characteristics

	Total (n = 357)	Responders (n = 234)	Non-responders (n = 123)	P value
Age, years	72.4 ± 7.9	71.9 ± 8.5	73.3 ± 6.3	0.095
Male	204(57.1)	138(59.0)	66(53.7)	0.335
BMI, kg/m²	24.7 ± 3.6	24.6 ± 3.9	24.9 ± 3.0	0.412
STS PROM score	6.7(5.6–7.2)	6.7(5.6–7.2)	6.7(5.7–7.3)	0.287
NYHA functional class III or IV	251(70.3)	160(68.4)	91(74.0)	0.270
NT-proBNP at baseline, pg/ml	1429.6(459.5–3416.0)	2103.5(930.0-4561.7)	421.0(252.0-1288.0)	< 0.001
NT-proBNP at discharge, pg/ml	852.0(357.3–1818.0)	707.6(302.0-1599.3)	1009.0(573.0-2543.0)	< 0.001
Prior history				
PCI	51(14.3)	30(12.8)	21(17.1)	0.275
CABG	12(3.4)	7(3.0)	5(4.1)	0.821
Stroke	43(12.0)	23(9.8)	20(16.3)	0.076
Permanent pacemaker	6(1.7)	6(2.6)	0(0.0)	0.097
Cancer	20(5.6)	11(4.7)	9(7.3)	0.307
Aortic valve surgery	14(3.9)	9(3.8)	5(4.1)	1.000
Comorbidities				
Hypertension	211(59.1)	121(51.7)	90(73.2)	< 0.001
Diabetes mellitus	84(23.5)	51(21.8)	33(26.8)	0.287
Hyperlipidaemia	138(38.7)	81(34.6)	57(46.3)	0.031
Chronic obstructive pulmonary disease	13(3.6)	8(3.4)	5(4.1)	0.990
Peripheral vascular disease	45(12.6)	20(8.5)	25(20.3)	0.001
Chronic heart failure	25(7.0)	22(9.4)	3(2.4)	0.014
Chronic kidney disease	12(3.4)	6(2.6)	6(4.9)	0.399
Liver disease	4(1.1)	3(1.3)	1(0.8)	1.000
Coronary artery disease	138(38.7)	92(39.3)	46(37.4)	0.724
Arrhythmia				
Atrial fibrillation	50(14.0)	36(15.4)	14(11.4)	0.300
Other type of arrhythmia	34(9.5)	19(8.1)	15(12.2)	0.213
Pure aortic insufficiency	29(8.1)	10(4.3)	19(15.4)	< 0.001
Echocardiographic assessment				
LVEF, %	60.0(50.0–65.0)	58.0(44.5–63.0)	63.0(60.0–68.0)	< 0.001
Maximum velocity, m/s	4.5(3.9–5.1)	4.6(4.0-5.2)	4.2(3.2–4.8)	< 0.001
Aortic regurgitation ≥ moderate	124(34.7)	78(33.3)	46(37.4)	0.443
Mitral regurgitation ≥ moderate	38(10.6)	32(13.7)	6(4.9)	0.010
Tricuspid regurgitation ≥ moderate	31(8.7)	28(12.0)	3(2.4)	0.002
Pulmonary hypertension	93(26.1)	77(32.9)	16(13.0)	< 0.001
Hospitalization length				
Intensive care unit (days)	1.0(1.0–3.0)	1.0(1.0–3.0)	1.0(1.0–2.0)	0.441
Total length (days)	12.0(9.0–17.0)	12.0(9.0–15.0)	14.0(11.0–19.0)	< 0.001

Values are mean ± SD, n (%), or median (interquartile range). BMI, body mass index; STS PROM, Society of Thoracic Surgeons Predicted Risk of Mortality; PCI, percutaneous coronary intervention; CABG, coronary artery bypass graft; NYHA, New York Heart Association; NT-proBNP, N-terminal pro–B-type natriuretic peptide; LVEF, left ventricular ejection fraction

### Procedural data and results

Procedural data and results are summarized in Table 2. No significant differences were observed between the responders and non-responders in terms of emergency surgery (4.8%), local anesthesia (16.6%), self-expandable valve (91.9%), post-procedure AR grade ≥ moderate (1.4%), conversion to open surgery (1.7%), and second valve implantation (19.6%). Of note, patients in the responder group were treated more frequently through transfemoral access (97.9% vs. 92.7%, p = 0.035).

**Table 2:** Procedural data and results

	Total (n = 357)	Responders (n = 234)	Non-responders (n = 123)	P value
Emergency surgery	17(4.8)	13(5.6)	4(3.3)	0.331
Local anesthesia	59(16.6)	40(17.1)	19(15.6)	0.714
Access				0.035
Transfemoral	343(96.1)	229(97.9)	114(92.7)	
Non-transfemoral (Carotid, Subclavian)	14(3.9)	5(2.1)	9(7.3)	
Valve type				0.223
Self-expandable valve (Venus-A, VitaFlow, TaurusOne)	328(91.9)	212(90.6)	116(94.3)	
Balloon-expandable valve (Sapien3, Sapien XT)	29(8.1)	22(9.4)	7(5.7)	
Post-procedure AR grade ≥ moderate	5(1.4)	2(0.9)	3(2.4)	0.345
Conversion to open surgery	6(1.7)	2(0.9)	4(3.3)	0.186
s valve implantation	70(19.6)	41(17.5)	29(23.6)	0.171
Device migration to ventricle	52(14.6)	29(12.4)	23(18.7)	
Paravalvular leakage	13(3.6)	8(3.4)	5(4.1)	
Device expand inadequately	5(1.4)	4(1.7)	1(0.8)	

Values are mean ± SD, n (%), or median (interquartile range)

### Clinical outcomes

**Fig. 1:**
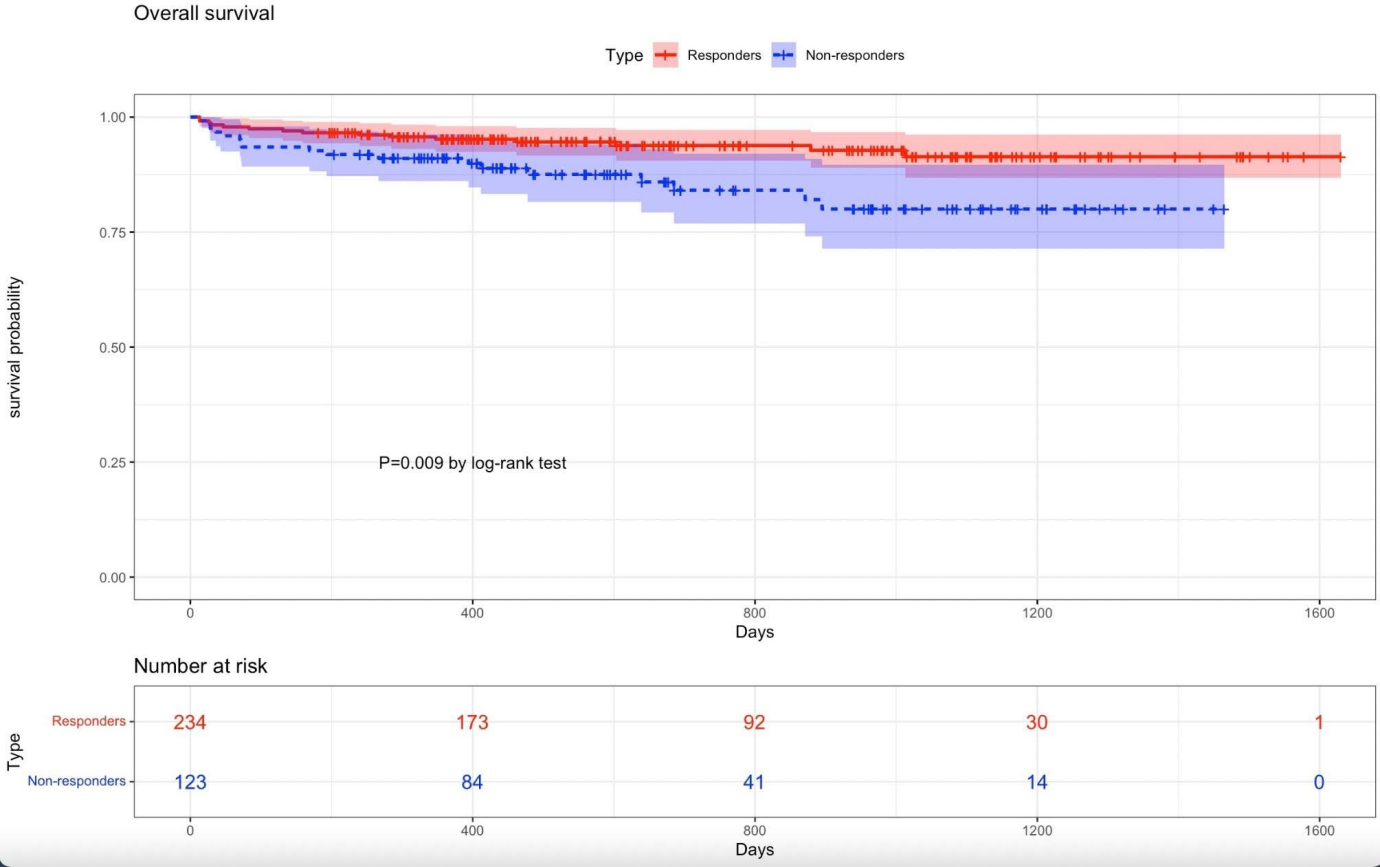
Kaplan-Meier survival curves for overall survival between NT-proBNP (N-terminal pro–B-type natriuretic peptide) responders and non-responders

VARC-3-related clinical outcomes are summarized in Table 3. There were no significant differences regarding 30-day all-cause mortality between the two groups (1.3% vs. 0.8, p = 0.689). However, patients in non-responder group were both significantly higher in cumulative all-cause mortality (20.0% vs. 8.6%, p = 0.009) and cardiac mortality (16.2% vs. 3.9%, p < 0.001) compared with patients in responder group. Kaplan-Meier survival curves stratified by NT-proBNP responders and non-responders are shown in Fig. 1. Rehospitalization for heart failure was no different in the two groups (13.2% vs. 8.7%, p = 0.334). Although all stroke were 19.5% in the responder group and 5.6% in the non-responder group, no significant difference was observed (p = 0.943).

**Table 3:** Clinical outcomes (Cumulative)

	Responders (n = 234)	Non-responders (n = 123)	P value by log-rank test
30-day all-cause mortality	3(1.3)	1(0.8)	0.689
Late all-cause mortality	15(8.6)	18(20.0)	0.009
Late cardiac mortality	7(3.9)	15(16.2)	< 0.001
Rehospitalization for heart failure	12(13.2)	9(8.7)	0.334
All stroke	12(19.5)	6(5.6)	0.943

Values are n (%)

### NT-proBNP in mortality prediction

In ROC analysis, NT-proBNP ratio significantly predicted cumulative all-cause mortality (AUC:0.631, 95%CI:0.526–0.736, p = 0.013; sensitivity 54.5%, and specificity 71.3% at a cutoff level of 1.114) and cardiac mortality (AUC:0.710, 95%CI:0.594–0.827, p = 0.001; sensitivity 68.2%, and specificity 71.3% at a cutoff level of 1.114). NT-proBNP at discharge also significantly predicted all-cause mortality (AUC:0.722, 95%CI:0.643–0.802, p < 0.001; sensitivity 72.7%, and specificity 68.5% at a cutoff level of 1350.5 pg/ml) and cardiac mortality (AUC:0.745, 95%CI:0.643–0.847, p < 0.001; sensitivity 81.8%, and specificity 69.3% at a cutoff level of 1396.5 pg/ml). However, NT-proBNP at baseline did not reach statistical significance in predicting cumulative all-cause mortality (AUC:0.569, 95%CI:0.475–0.663, p = 0.192) or cardiac mortality (AUC:0.512, 95%CI:0.398–0.627, p = 0.847).

Multivariable logistic regression analysis revealed that baseline NT-proBNP ≥ 1429.60 pg/ml (odds ratio [OR]: 0.257, 95% CI: 0.136 to 0.487, p < 0.001), peripheral vascular disease (OR: 2.537,95%CI:1.091 to 5.971, p = 0.031), left ventricular ejection fraction (OR: 1.042, 95%CI: 1.010 to 1.075 per 1%, p = 0.011), maximum velocity (OR:0.576, 95%CI: 0.414 to 0.801 per 1 m/s, p = 0.001) were independent determinants of NT-proBNP non-responders (Table 4).

**Table 4:** Predictors of NT-proBNP non-responders

Variables	Adjusted OR	95% CI	P value
Hypertension	1.637	0.922–2.905	0.092
Hyperlipidaemia	1.154	0.661–2.016	0.613
NT-proBNP ≥ 1429.60 at baseline	0.257	0.136–0.487	< 0.001
Peripheral vascular disease	2.537	1.091–5.971	0.031
Chronic heart failure	0.581	0.128–2.643	0.482
Pure aortic insufficiency	0.957	0.277–3.305	0.945
Left ventricular ejection fraction, increase by 1%	1.042	1.010–1.075	0.011
Maximum velocity, increase by 1 m/s	0.576	0.414–0.801	0.001
Mitral regurgitation ≥ moderate	1.114	0.359–3.454	0.851
Tricuspid regurgitation ≥ moderate	0.239	0.052–1.098	0.066
Pulmonary hypertension	0.742	0.350–1.570	0.434
Total length of hospitalization, increase by 1 day	1.031	1.000-1.063	0.053

OR, odds ratio; CI, confidence interval. NT-proBNP, N-terminal pro–B-type natriuretic peptide

Univariable and multivariable Cox regression analysis was used to identify variables that show statistically significant associations with all-cause mortality during follow-up period. These data are summarized in Table 5. Predictors of all-cause mortality in descending order of HR were NT-proBNP non-responders (HR:4.779, 95%CI:1.815–12.587, p = 0.002), NT-proBNP ≥ 1350.5 pg/ml at discharge (HR:4.218, 95%CI:1.340-13.279, p = 0.014), mitral regurgitation ≥ moderate (HR:3.064, 95%CI:1.046–8.973, p = 0.041), coronary artery disease (HR:2.974, 95%CI:1.131–7.821, p = 0.027), length of intensive care unit (HR:1.051, 95%CI:1.018 to 1.084 per 1 day, p = 0.002) according to multivariable Cox analysis. A univariate Cox regression analysis was conducted to show the significant differences of clinical outcomes among patients with NT-proBNP ≥ 1350.5 pg/ml at discharge according to the NT-ProBNP ratio in Supplementary Table 1. It reveals that NT-proBNP non-responders significantly predicted the late all-cause mortality (HR: 3.061, 95%CI: 1.309–7.154, p = 0.010) and late cardiac mortality (HR: 3.977, 95%CI: 1.417–11.159, p = 0.009).

**Table 5:** Cox regression analysis for late all-cause mortality

Variable	Univariate		Multivariate	
	HR (95%CI)	P value	HR (95%CI)	P value
NT-proBNP non-responders	2.434(1.226–4.830)	0.011	4.779(1.815–12.587)	0.002
Coronary artery disease	3.169(1.536–6.537)	0.002	2.974 (1.131–7.821)	0.027
Prior stroke	2.593(1.201–5.597)	0.015	——	——
Pure aortic insufficiency	2.506(1.034–6.072)	0.042	——	——
Length of hospitalization, increase by 1 day	1.039(1.022–1.057)	< 0.001	——	——
Intensive care unit, increase by 1 day	1.062(1.036–1.088)	< 0.001	1.051(1.018–1.084)	0.002
Mitral regurgitation ≥ moderate	2.449(1.062–5.647)	0.036	3.064(1.046–8.973)	0.041
Maximum velocity, increase by 1 m/s	0.726(0.554–0.951)	0.020	——	——
NT-proBNP ≥ 1350.5 pg/ml at discharge	4.864(2.260-10.468)	< 0.001	4.218(1.340-13.279)	0.014

NT-proBNP, N-terminal pro–B-type natriuretic peptide; HR, hazard ratio; CI, confidence interval

**Fig. 2:**
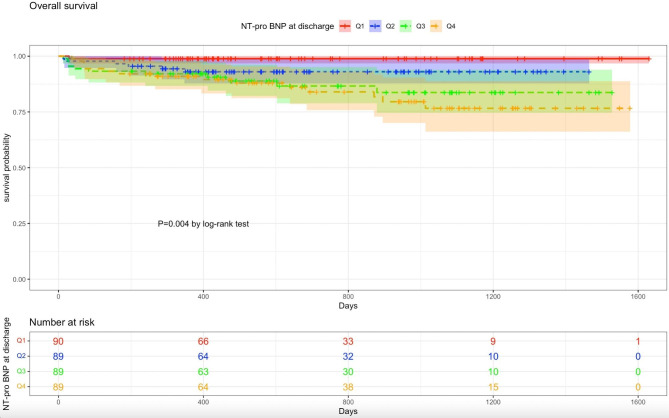
Kaplan-Meier survival curves for overall survival of subgroups based on NT-proBNP (N-terminal pro–B-type natriuretic peptide) levels at hospital discharge. (Q4 = upper quartile). Q indicates quartile

Kaplan-Meier survival curves stratified by quartiles of NT-proBNP at discharge are shown in Fig. 2. Survival curves of subgroups of NT-proBNP at discharge substantially diverged, illustrating patients with high NT-proBNP levels at discharge (upper quartile) showed the worst survival (log-rank test, p = 0.004). The Cochrane-Armitage test was used to identify the trend of late all-cause mortality across quartiles of NT-proBNP levels at discharge, revealing a significant increase in all-cause mortality as NT-proBNP at discharge increased (p < 0.001).

## Discussion

In this study, we examined the association between periprocedural changes of NT-proBNP levels and clinical outcomes after TAVR based on NT-proBNP ratio (postprocedural NT-proBNP at discharge/ preprocedural NT-proBNP). Patients were grouped as responders (NT-proBNP ratio < 1) and non-responders (NT-proBNP ratio ≥ 1). The major findings of the present study were as follows:

1. Median NT-proBNP levels decreased from 1429.6 pg/ml at baseline to 852.0 pg/ml at discharge.
2. 34.5% of the study patients were non-responders.
3. Baseline NT-proBNP ≥ 1429.60 pg/ml, peripheral vascular disease, left ventricular ejection fraction, maximum transvalvular velocity were independent determinants of NT-proBNP non-responders.
4. NT-proBNP ratio and NT-proBNP at discharge significantly predicted cumulative all-cause and cardiac mortality after TAVR.
5. NT-proBNP non-responders, NT-proBNP ≥ 1350.5 pg/ml at discharge, baseline mitral regurgitation ≥ moderate, coronary artery disease, and length of intensive care unit were independently associated with lower survival rates during follow-up time.
6. As levels of NT-proBNP at discharge increased, all-cause mortality significantly increased after TAVR.

NT-proBNP has been identified as a diagnostic and prognostic biomarker for a number of cardiovascular diseases [15]. The utility of NT-proBNP levels for risk stratification and as a predictor of adverse clinical outcomes after TAVR has been studied [16, 17]. Studies analyzing the predictive value of periprocedural changes of NT-proBNP in TAVR have yielded conflicting results. In most studies, baseline NT-proBNP levels were identified as predictors for short- and long-term mortality after TAVR [16, 18, 19]. In contrast, other studies found no correlation between baseline NT-proBNP and postprocedural mortality [20]. In our study, no significant association were observed between baseline NT-proBNP levels and all-cause mortality after TAVR. Besides, we found that postprocedural survival rates of NT-proBNP responders and patients with lower NT-proBNP at discharge were better.

Of note, the NT-proBNP responders had higher baseline NT-proBNP levels, lower left ventricular ejection fraction, higher maximum transvalvular velocity and more patients with chronic heart failure. This finding was consistent with previous observation that TAVR in this patient group produced immediate effects in left ventricular unloading and decrease of myocardial stretch, resulting in an acute decrease in circulating NT-proBNP levels after TVAR. An intuitive hypothesis can be made from our findings that patients whose elevated NT-proBNP levels are primarily driven by severe AS and heart failure would benefit most from TAVR.

The association of periprocedural changes of NT-proBNP with survival after TAVR was investigated by Seoudy et al. [21] The study measured NT-proBNP in 704 patients before TAVR and at discharge. The authors demonstrated that highly elevated NT-proBNP levels—both at baseline and at discharge are significantly associated with survival regardless of a postprocedural decrease of NT-proBNP after TAVR. However, NT-proBNP at baseline was not directly associated with all-cause mortality in our study. In our patient population, NT-proBNP at baseline ≥ 1429.60 pg/ml suggested lower non-responder risk, leading to reduce overall and cardiac mortality rates. Unfortunately, end points such as cardiac mortality and rehospitalization for heart failure were not accounted by Seoudy et al. We found that NT-proBNP responders were significantly associated with lower cardiac mortality, but were not associated with rehospitalization for heart failure.

Given the adverse correlation between the increase of NT-proBNP level after TAVR and the poor clinical outcomes, two questions are raised: How to alleviate the increase of NT-proBNP level after TAVR? Will the efforts aimed at pathobiology driving the increase of NT-proBNP after TAVR improve the clinical outcomes? It needs to be clear that the potential therapeutic targets are underlying pathobiology, such as maladaptive hypertrophy, abnormal left ventricular function, increased wall stress, and volume overload, which lead to NT-proBNP release [22]. Optimal medical therapy may mitigate the elevations in NT-proBNP after TAVR. This hypothesis is warranted to confirm in future strategy trials, and whether targeting lower postprocedural NT-proBNP levels may improve clinical outcomes needs to be assessed in future studies.

It is worth using NT-proBNP for risk stratification of TAVR patients, identifying NT-proBNP non-responders earlier, and reducing postprocedural circulating NT-proBNP levels by more optimal timing of TAVR procedure and medical management, which are of great help in improving the prognosis of TAVR patients.

### Strengths and limitations

The study is limited inherently by its retrospective single-center design. Data on medical management were not systematically collected in this study. Serial changes of NT-proBNP were not measured in all patients after hospital discharge. The external validation is needed as for the cut-off value of NT-proBNP at baseline or discharge. The present study showed that about 20% of patients experienced second valve implantation mainly due to device migration to left ventricle. This might be explained by the immaturity of early technology, the characteristics of prosthesis, and the cardiac anatomical characteristics of patients. However, our study cohort represents a typical, unselected, real-world TAVR population with a considerable sample size.

## Conclusions

Periprocedural changes of NT-proBNP is associated with cumulative all-cause and cardiac mortality after TAVR. NT-proBNP ratio and NT-proBNP at discharge should be carefully monitored to improve postprocedural risk stratification and guide optimal treatment strategy selection.

### Electronic supplementary material

Below is the link to the electronic supplementary material.

Supplementary Material 1

## Data Availability

The data that support the findings of this study are available from the corresponding author upon reasonable request.
