# Supplementary material for: N-terminal pro-B-type natriuretic peptide is associated with clinical outcomes after transcatheter aortic valve replacement
Source: J Cardiothorac Surg. 2023 Oct 10;18:286. doi: 10.1186/s13019-023-02391-2 (PMC10566171; doi:10.1186/s13019-023-02391-2)
Supplement: Supplementary file 1 — Supplementary Material 1 [file 13019_2023_2391_MOESM1_ESM.docx]

Supplementary Table 1. Clinical outcomes among patients with NT-proBNP ≥1350.5 pg/ml at discharge according to the NT-ProBNP ratio.

| Clinical outcomes | Univariate | |
| --- | --- | --- |
|  | HR (95%CI) | P value |
| Late all-cause mortality | 3.061(1.309-7.154) | 0.010 |
| Late cardiac mortality | 3.977(1.417-11.159) | 0.009 |
| Rehospitalization for heart failure | 2.165(0.611-7.675) | 0.232 |
| All stroke | 6.994(0.817-59.872) | 0.076 |

NT-proBNP, N-terminal pro–B-type natriuretic peptide; HR, hazard ratio; CI, confidence interval.
